# Radiofrequency currents exert cytotoxic effects in NB69 human neuroblastoma cells but not in peripheral blood mononuclear cells

**DOI:** 10.3892/ijo.2012.1569

**Published:** 2012-07-24

**Authors:** MARÍA LUISA HERNÁNDEZ-BULE, ERNESTO ROLDÁN, JOAQUÍN MATILLA, MARÍA ÁNGELES TRILLO, ALEJANDRO ÚBEDA

**Affiliations:** 1 Departments of Research-BEM-IRYCIS; 2 Clinical Immunology, Cellular and Tumoral Immunology Section and; 3 Research-Biochemistry-IRYCIS, Hospital Ramon y Cajal, 28034 Madrid, Spain

**Keywords:** electric therapy, neuroblastoma, peripheral blood mono-nuclear cells, cell cycle, cytostasis

## Abstract

Recently, a number of electric and electrothermal therapies have been applied to the treatment of specific cancer types. However, the cellular and molecular mechanisms involved in the response to such therapies have not been well characterized yet. Capacitive-resistive electric transfer (CRET) therapy uses electric currents at frequencies within the 0.45–0.6 MHz range to induce hyperthermia in target tissues. Preliminary trials in cancer patients have shown consistent signs that CRET could slow down growth of tumor tissues in brain gliomas, without inducing detectable damage in the surrounding healthy tissue. Previous studies by our group have shown that subthermal treatment with 0.57-MHz electric currents can induce a cytostatic, not cytotoxic response in HepG2 human hepatocarcinoma cells; such effect being mediated by cell cycle alterations. In contrast, the study of the response of NB69 human neuroblastoma cells to the same electric treatment revealed consistent indications of cytotoxic effects. The present study extends the knowledge on the response of NB69 cells to the subthermal stimulus, comparing it to that of primary cultures of human peripheral blood mononuclear cells (PBMC) exposed to the same treatment. The results showed no sensitivity of PBMC to the 0.57 MHz subthermal currents and confirmed that the treatment exerts a cytotoxic action in NB69 cells. The data also revealed a previously undetected cytostatic response of the neuroblastoma cell line. CRET currents affected NB69 cell proliferation by significantly reducing the fraction of cells in the phase G_2_/M of the cell cycle at 12 h of exposure. These data provide new information on the mechanisms of response to CRET therapy, and are consistent with a cytotoxic and/or cytostatic action of the electric treatment, which would affect human cells of tumor origin but not normal cells with a low proliferation rate.

## Introduction

A variety of electrical therapies applying different signal types and treatment protocols are currently used in clinical practice. Electrotherapy has been reported effective in the treatment of pain, blood flow promotion, decreased tone of skeletal striated muscle, metabolism acceleration and resorption of edema and joint effusion (1,2). Also, some electrotherapeutic techniques have been assayed recently for treatment of different cancer types (3–7). The present study investigates the cellular response to electrical stimuli currently used in electrothermal therapies that apply the capacitive-resistive electric transfer (CRET) technique. This non-invasive therapy is based on localized hyperthermia induced in inner tissues when exposed to sine wave electric currents with frequencies between 0.45 and 0.60 MHz. Such currents, which are delivered through movable electrode pairs applied to the epidermis, induce a thermal Joule's effect in the targeted organs due to the electric resistivity of the tissues (8).

Preliminary assays of CRET therapy on cancer patients showed consistent signs of slowing down the growth of the tumor tissues, without adversely affecting the surrounding healthy tissues (9–11). Similar effects have been reported in response to other therapies based on capacitive hyperthermia (12–14). Although these and other medical effects of electro-thermal therapies have been widely described, the cellular and molecular mechanisms involved in the biological responses to electric currents applied by such therapies have only recently begun to be studied (15–19).

The clinical and experimental evidence suggests that the therapeutic action of the CRET currents within the 0.45–0.60 MHz spectrum, might be exerted not only through heating of the treated tissues, but also by a direct, cellular or tissue response to the electric stimuli. It is indicated by previously reported results that exposure to 0.57–MHz currents at subthermal densities can induce cytostasis in the human hepatocarcinoma cell line HepG2 (16). Such effect is expressed as a decline in cell proliferation, induced by changes in expression and activation of cell cycle control proteins, cyclins D1, A and B1, and of the cell cycle inhibitor protein p27^Kip1^(17). These data indicate that the cytostatic action of CRET currents applied to subthermal doses could be mediated by alterations in cell cycle regulation.

On the other hand, preliminary data indicate that stimulation with the above described electric parameters could exert a partial cytotoxic action on the human neuroblastoma cell line NB69, whose proliferation rate is higher than that of the HepG2 line. This cytotoxic response is expressed as an increased rate of cell death, accompanied with changes in the levels of total proteins and DNA in the cultures (15). The aim of the present work is to study the nature of the cytotoxic response observed in the neuroblastoma cell line, investigating whether the electric stimulation can deregulate the cell cycle of NB69 by inducing changes similar to those described in the line HepG2 (16,17). Additionally, it is important to elucidate whether subthermal stimulation with CRET signal can induce in normal, nontumoral human cells, cytotoxic or cytostatic responses similar to those described in human cancer cells. In this regard, it is of particular interest to determine whether the electric stimulation could alter the viability of immune cells, since they can participate in cancer control through tumor antigen recognition. Consequently, this work further addresses characterization of the cellular response to CRET currents by studying their potential effects on primary cultures of peripheral blood mononuclear cells (PBMC) from healthy donors.

The obtained results support our preliminary data, confirming that subthermal treatment with 0.57 MHz currents induces a partial cytotoxic response in NB69 cells. Also, a cytostatic response potentially due to alterations in cell cycle kinetics was observed. These results, together with the previously reported effects on the HepG2 cells, suggest that treatment with subthermal doses of 0.57 MHz could exert antiproliferative effects on two separate human cell lines of cancer origin. Such effects appear to be due to interference of the electric stimulus with mechanisms involved in the cell cycle regulation of both cell lines. By contrast, viability of PBMC from healthy donors was not affected by exposure to the same electric parameters. As a whole, our results support the hypothesis that the therapeutic action of thermoelectric treatments of the CRET type is mediated, at least in part, by a direct cellular response to the electric stimulus, being the proliferative lines more sensitive than the non-proliferating PBMC. The effect at the cellular level could act in synergy with the thermal action at the tissue level, exerted by the current when passing through tissues with a relatively high electric resistivity.

## Materials and methods

### Cell cultures

Cells of the human neuroblastoma line NB69 were maintained in D-MEM supplemented with 15% (vol/vol) foetal bovine serum (FBS), L-glutamine (4 mM) and penicillin-streptomycin with Fungizone (100 U/ml) (all from Gibco, Paisley, UK). Cells were seeded in 60 mm Ø Petri dishes (Nunc, Roskilde, Denmark), at a 5x10^4^ cells/ml. Ten total dishes per experimental replicate were seeded, five for treatment and five controls, and maintained in culture for 4 days with 5% CO_2_ at 37°C. The culture medium was renewed every 3 days. For BrdU assay and immunocytochemical analysis, cells were seeded on coverslips (Ø 12 mm) placed inside the Petri dishes.

Buffy coat from whole blood obtained from healthy donors was used as the source of PBMC. Samples from ten donors were used. PBMC were isolated by centrifugation in Ficoll-Hypaque density gradient (Lymphoprep, Nycomed, Zurich, Switzerland), using the method described by Boyum in 1968 (20), and maintained in RPMI supplemented with 10% fetal bovine serum, 1% L-glutamine, 0.1% gentamicine (all from Gibco) and 1% penicillin-streptomycin (ICN, OH, USA). Once isolated, the PBMC were seeded on 60 mm Ø Petri dishes (Corning NY, USA) at a density of 2.75x10^6^ cells/ml. Twenty total dishes were seeded per experimental replicate, ten dishes for treatment and ten controls. Before stimulation, cultures were incubated for 1 h in a 5% CO_2_ atmosphere at 37°C.

### Exposure system and experimental protocol

**Figure 1:**
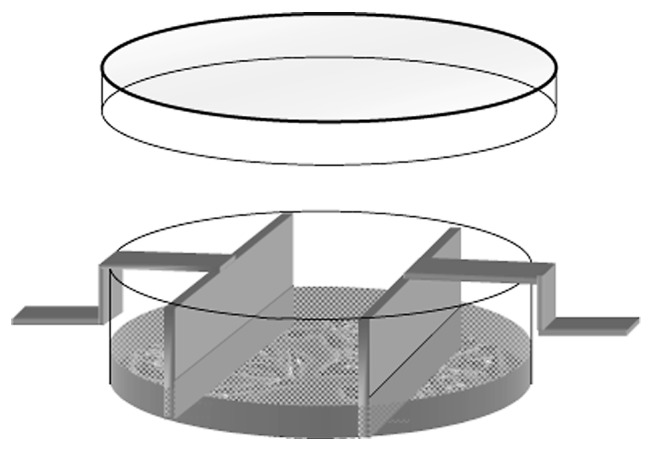
Diagram showing a set of stainless steel electrodes for stimulation of a cell culture in a Petri dish.

**Figure 2:**
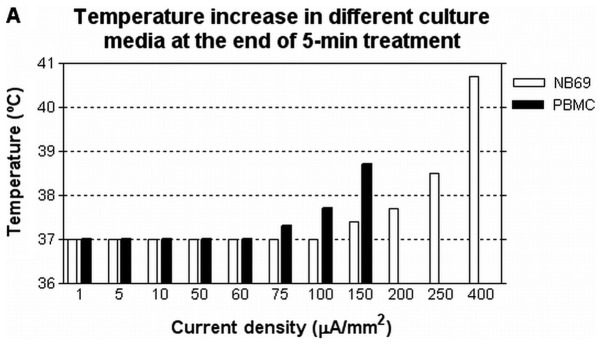

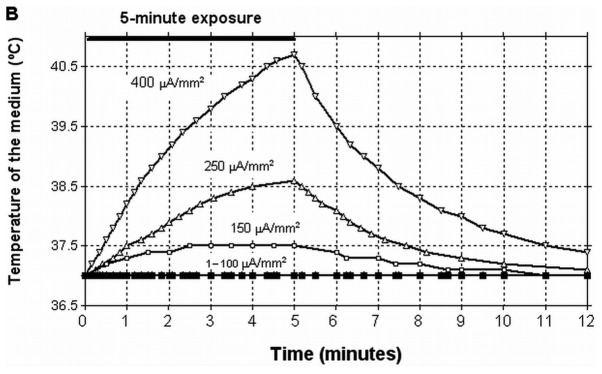
(A) Temperature reached by the culture media for NB69 and PBMC at the end of a 5-min pulse, as a function of the current density. Digital thermometers HIBOX 16 (Hibox, Taiwan) were used to register the media temperature after 5-min exposure to currents densities ranging 1–400 μA/mm^2^. Current densities j ≥150 μA/mm^2^ induced temperature increase in the NB69 culture medium. In the PBMC medium, thermal increase occurred at values j ≥75 μA/mm^2^. The current density j = 50 μA/mm^2^ was selected to study the responses of both cell types to a subthermal stimulus. (B) Evolution of the temperature in the NB69 culture medium during 5 min of electric stimulation with different current densities, and during 7 min of post-exposure. Same procedure as in (A).

The exposure system has been described in detail in previous reports (16,17). Briefly, the exposure to 0.57 MHz electric currents was carried out through pairs of sterile stainless steel electrodes, designed *ad hoc* for *in vitro* stimulation, inserted inside each of the control and exposed Petri dishes (Fig. 1). In the experimental groups, the electrode pairs were connected in series to a signal generator (model M-500, INDIBA S.A., Barcelona, Spain). The stimulation pattern consisted of a 5-min pulse of 0.57 MHz, sine wave currents, delivered every 4 h along a 24-h period. The current density values to be tested were selected from recordings of the temperature induced by the electric current flowing through the culture medium (Fig. 2). In the present work, we studied the viability and proliferation of NB69 cells stimulated with different subthermal current densities, between 1 and 100 μA/mm^2^, and with a thermal density of 400 μA/mm^2^. For PBMC assays, only the 50 μA/mm^2^ current density, which had proven effective in our previously published studies, was applied. The signal parameters as well as temperature, relative humidity and CO_2_ partial pressure into incubators were monitored during experiments. In NB69 assays, the electric treatment started at day four post-seeding, during exponential cell growth phase, and finished 24 h after, at day five post-seeding. To study the chronology of the NB69 response, analyses were carried out at the beginning of exposure (0 h), during the treatment (at 6 or 12 h), at the end of treatment (24 h) and after two days of post-exposure incubation (72 h). For PBMC, after 1 h of post-seeding incubation, the cell cultures were electrically stimulated with 5-min pulses for 24 h. At the end of treatment the cells were harvested for analysis. All experimental procedures were carried out in blind condition for treatment.

### Artifact control

To verify that the cellular responses obtained were induced exclusively by the electric stimuli, and not by other, uncontrolled environmental factors, assays were conducted on the influence of the non-energized electrodes on cell viability. Also, a number of potentially influencing factors were studied, including the electrochemical integrity of the electrodes, electrophoretic effects, electromagnetic fields and thermal effects. The procedures applied have been described in previous studies (16) and the data (not shown) confirmed that none of the studied factors changed significantly either the physical/chemical properties of the culture medium or the cellular behaviour.

### Effects on the cell viability of NB69 cultures

The effect of 1, 5, 10, 50, 100 or 400 μA/mm^2^ stimuli on the cell viability of NB69 was studied through dye exclusion with 0.4% trypan blue (Sigma, Steinheim, Germany) at the end of the electric treatment. Three experimental replicates were conducted for each of the assayed current densities.

### Effects on cellular viability of PBMC culture

For PBMC, samples stimulated with 50 μA/mm^2^ and their respective controls were harvested at the end of 24 h of intermittent treatment. Cell viability of the PBMC cultures was quantified by trypan blue exclusion method, following the same protocol as for NB69. Ten independent analysis were carried out, one for each of the obtained blood samples. MTT assay (Roche, Indianapolis, IN, USA) was applied following the manufacturer's instructions, to confirm the trypan blue data. MTT quantification was carried out on samples from two different donors, using ELISA reader at 600 nm. As complementary assay, and in order to study the cell density, the total protein content of the cultures was quantified through the Lowry method, following protocols described in previous reports (15,16). Seven independent samples from seven different donors were analyzed.

### BrdU assay of NB69

On the basis of our previous data on the responses electrically induced in lines NB69 and HepG2 (16,17), a current density of 50 μA/mm^2^ was chosen to conduct this assay. The effects of the exposure on cell proliferation of NB69 were analyzed through 5-bromo-2′-deoxyuridine assay, following the protocol described by Hernández-Bule *et al*(16). Briefly, just before stimulation onset (0 h), the dishes were incubated with 5-bromo-2′-deoxyuridine (Dako, Denmark) at a 5x10^−4^ M concentration. At the end of the 24 h of exposure and/or incubation, the cells were fixed with 4% paraformaldehyde (Merck, Darmstadt, Germany) and incubated with monoclonal primary antibody anti-BrdU (Dako) and anti-mouse Ig flourescein-linked whole antibody as secondary antibody (Amersham Biosciences, Buckinghamshire, UK). The preparations were counterstained and mounted in glycerol with Hoechst 33342 fluorescence dye (Bisbenzimide, Sigma) and studied through fluorescence microscopy (Nikon Eclipse TE300; Melville, USA) and Computer-Assisted Image Analysis (Analy-SIS, GMBH, Munich, Germany). Three experimental replicates were carried out, and about 7200 cells were studied per experimental group.

### PCNA immunofluorescence of NB69

The proliferating cell nuclear antigen (PCNA) was used to estimate potential changes induced in DNA content at specific time intervals. Cells were grown on 12-mm Ø coverslips placed on the bottom of the Petri dishes, on the surface between electrodes. The samples treated with 50 μA/mm^2^ and their controls were fixed with 4% paraformaldehyde (Merck). The cells were permeabilized and incubated overnight at 4°C with primary monoclonal antibody anti-PCNA (Santa Cruz Biotechnology, Santa Cruz, CA, USA). After washing, the cells were incubated with secondary antibodies conjugated to Alexa Green dye (Molecular Probes, CA, USA), counterstained and mounted in glycerol with Hoechst 33342 (Sigma). The samples were analyzed through fluorescence microscopy (Nikon) and computer-assisted image analysis (Analy-SIS, GMBH). Four experimental repeats were conducted and two coverslips were analyzed per experimental group, one for each of the studied time intervals (0, 12 or 24 h). The percent of PCNA-positive cells and the total nuclei (revealed with Hoechst 33342, Sigma) were recorded in 15 randomly selected fields per coverslip, and a total of ∼5,000 cells per experimental group were evaluated.

### Flow cytometric analysis of effects on apoptosis and cell cycle of NB69

After 6, 12 or 24 h of 50 μA/mm^2^ exposure and/or incubation, neuroblastoma samples were fixed with 70% ethanol as described in previous studies (16). The relative fractions of cellular subpopulations in different phases of the cell cycle, including the sub-G_1_ population (cells with less than stationary cell complement of DNA), a qualitative indicator of apoptotic cells, were determined. A minimum of 2 experimental replicates were conducted for each of the three temporal intervals analyzed. The cell cycle kinetics was determined by CellQuest 3.2 software (Becton-Dickinson).

### Flow cytometric analysis of PBMC subpopulations

In order to detect potential, electrically-induced changes in the relative rates of T lymphocytes, B lymphocytes, natural killer cells and cells with monocytic phenotype, antibodies anti-CD14 linked to phycoerytrin (PE), anti-CD36 linked to fluorescein isothiocyanate (FITC), anti-CD56 linked to phycoerytrin and anti-CD3 linked to allophycocyanin (APC) (all from Becton-Dickinson, Franklin Lakes, NJ, USA) were used. The B lymphocyte fraction was estimated by exclusion of the enumerated subpopulations. Cell suspension samples (1x10^6^ cells per sample) were collected after 24 h of sham or intermittent stimulation with 50 μA/mm^2^ and incubated with the corresponding antibodies, in the dark and at room temperature, for 30 min. Flow cytometry of PBMC from two donors was carried out using Becton-Dickinson FACScan (FACScalibur, Becton-Dickinson). Twenty thousand events (cells) per cell suspension were analyzed.

### Statistical analysis

Data were analyzed using unpaired, two-tailed Student's t-test. Differences p<0.05 were considered statistically significant.

## Results

### Effects on NB69 cell viability as a function of current density

**Figure 3:**
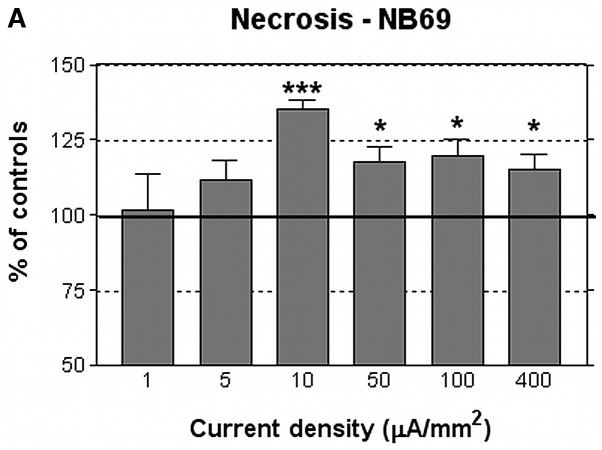

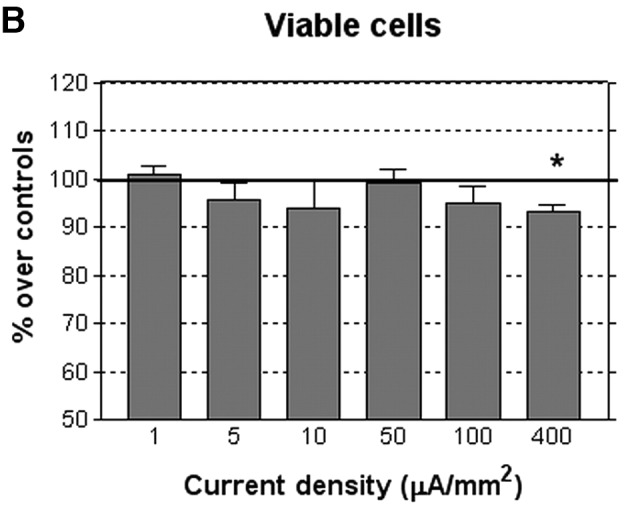
(A) Rate of necrosis in NB69, quantified by trypan blue dye. Necrotic cells after 24 h of stimulation with different current densities. (B) Viability rate in NB69, quantified by trypan blue dye. Viable cells after 24 h of stimulation with different current densities. Data are normalized over the corresponding control samples. Means ± SEM of three or four replicates for each current density tested, with 5 exposed dishes and 5 controls per replicate and current density. ^***^p<0.001; ^*^0.01< p<0.05, Student's t-test.

In the assayed culture conditions, the average rate of necrosis in the control NB69 samples after 24 h of incubation in the presence of electrode pairs was 14±1%. This survival rate corresponds to that classically reported in the literature for NB69 cells incubated in normal, control conditions (21). At the end of the 24 h of intermittent treatment with current densities j ≥10 μA/mm^2^, the rate of necrosis had increased significantly, by 18–35% over that of the corresponding controls samples (Fig. 3A). This cytotoxic effect was not linearly related to the current density. In fact, the 400 μA/mm^2^ current, which as shown in Fig. 2B, increased by 3.5°C the temperature of the medium at the end of each 5-min exposure pulses, induced cytotoxicity rates equal or below those induced by weaker densities. Densities j ≤5 μA/mm^2^ did not induce significant changes in the rate of necrosis when compared to controls. Regarding the rate of viability of treated cultures, in general it was slightly reduced, ∼7% below controls. The effect reached statistical significance only for the thermal dose of 400 μA/mm^2^ (Fig. 3B).

### Effects of the 50 μA/mm^2^ electric current on DNA synthesis in NB69

**Figure 4:**
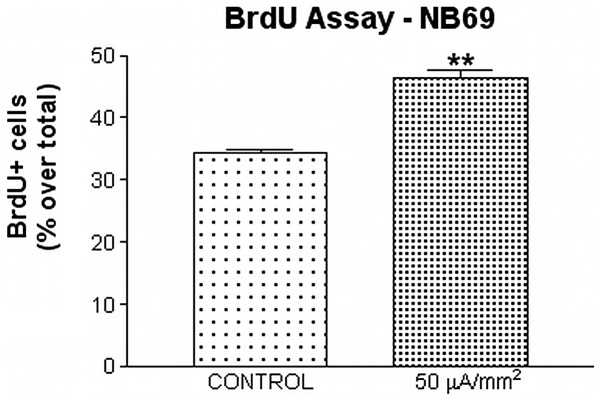
BrdU assay. BrdU incorporation rate in NB69 samples after 24 h of intermittent treatment with 50 μA/mm^2^ current density. Percent of BrdU-labeled cells in the total sample. Approximately 15,000 cells were counted in a total of three experimental replicates. Approximately 7,200 cells per experimental group were evaluated. Means ± SEM; ^**^0.001< p<0.01, Student's t-test.

**Figure 5:**
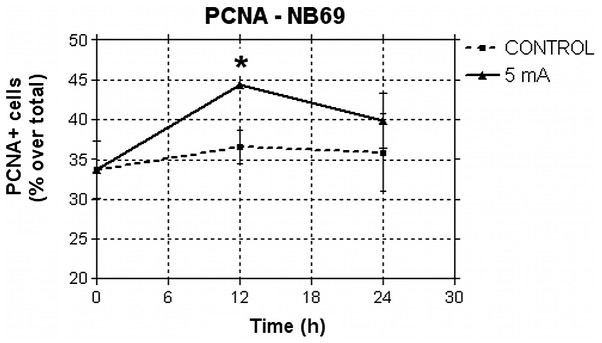
Immunofluorescence assessment of PCNA expression in NB69. Percents of PCNA-positive cells before (t = 0 h), during (t = 12 h) and the end (t = 24 h) of treatment with 50 μA/mm^2^. The fraction of cells undergoing DNA synthesis increased significantly during treatment. Approximately 23,000 cells were counted in a total of four experimental replicates. Approximately 5,000 cells per experimental group were evaluated. Means ± SEM; ^*^0.01< p<0.05, Student's t-test.

Our previous studies have reported that hepatocellular carcinoma cells are sensitive to exposure to 50 μA/mm^2^ CRET currents (17). From those data and from the results described in the preceding paragraph on NB69 cell viability at the end of the electric treatment, it was decided that all further trials would be conducted at a current density of 50 μA/mm^2^. DNA synthesis was quantified through two complementary techniques: quantification of BrdU incorporation during the 24 h of intermittent treatment, and study of the levels of expression of proliferating cell nuclear antigen PCNA before (0 h), during (12 h) and at the end of the exposure period (24 h). The data showed that 24 h of treatment with the 50 μA/mm^2^ current significantly increased BrdU incorporation in NB69 cells (35.08% over controls; 0.001<p<0.01; Fig. 4), which indicates that the electric stimulus enhances DNA synthesis. The immunofluorescence assay for PCNA reinforced this evidence by showing a significant increase (21.49% over controls, 0.01 < p<0.05) in the percent of cells expressing this antigen at 12 h after treatment onset. The increased expression of PCNA seemed to last until the end of 24 h of treatment (11.14% over controls), although at that point the differences between the stimulated samples and their controls did not reach statistical significance (Fig. 5).

### Effects of the 50 μA/mm^2^ electric current on the cell cycle of NB69

**Figure 6:**
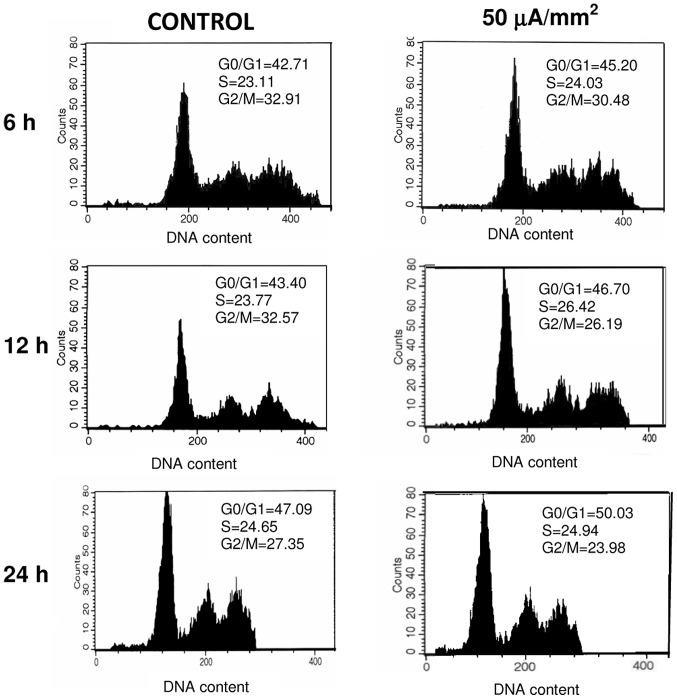
Flow cytometric analysis of cell cycle phases in NB69 samples exposed to the 50 μA/mm^2^ stimulus. Cells were collected during (6 and 12 h) and at the end of the treatment (24 h) and stained with propidium iodide for flow cytometry analysis. A minimum of 2 experimental replicates were conducted in each three time periods. The image shows representative results obtained from one of the replicates. Each histogram represents the analysis of 20,000 events obtained from the corresponding samples.

As described above, the rate of cell death in NB69 was significantly increased at the end of the 24-h intermittent stimulation (Fig. 3A). On the other hand, the treatment also induced an increase in DNA synthesis through BrdU incorporation (Fig. 4) as well as increased rate of PCNA-positive cells (Fig. 5), which could be indicative of a cytoproliferative response. To elucidate the causes of such apparent contradiction, we studied the cell cycle through flow cytometry, during the treatment (6 and 12 h after the exposure onset) and at the end of the treatment (24 h). The results of the analysis showed a modest increase, non-significant statistically, of cells in S phase after 6 h of exposure, when compared to the corresponding sham samples (Fig. 6 and Table I). After six additional hours of treatment, the fraction of the cellular population in S phase did not differ from that of the control samples, but a statistically significant decrease in the rate of cells in G_2_/M (17.9% below controls, 0.01< p<0.05) was detected in the exposed group. At the end of 24 h of treatment no significant differences were detected with respect to controls. These results may indicate that the electric stimulation induced either a blocking of the cell cycle in S phase, or a prolongation of S phase and, hence, of the time necessary to complete a cycle. This is consistent with the peak of DNA synthesis observed at 12 and 24 h of treatment, as revealed both by PCNA immunofluorescence and BrdU incorporation (Figs. 4 and 5).

Besides, the graphs in Fig. 6 revealed absence of the DNA sub-G_1_ peak, a qualitative indicator of apoptosis. This suggests that the electric treatment does not induce apoptosis during the studied intervals. Additional, more specific tests and a study of apoptosis after the treatment would be needed to confirm these indications.

### Effects of the 50 μA/mm^2^ electric current on the viability of PBMC

**Figure 7:**
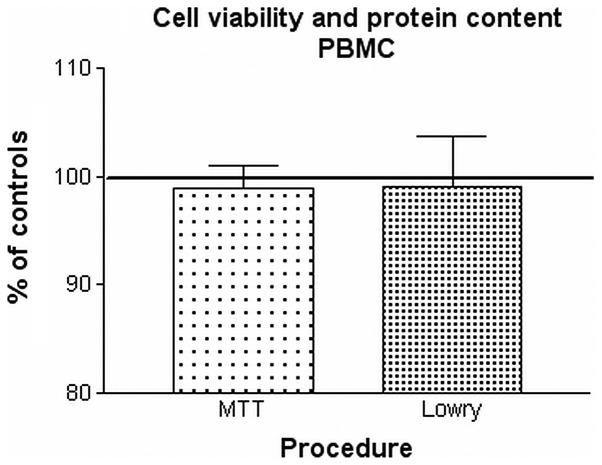
Cell viability and protein content of PBMC after 24-h treatment with 50 μA/mm^2^. Comparison of the response through MTT method (two experimental replicates) and Lowry method for protein spectrophotometry (mg of protein per dish in seven experimental replicates). Means ± SEM of data normalized over controls. Differences with respect to controls were not statistically significant.

In contrast to the response of neuroblastoma cells, exposure to 50 μA/mm^2^ did not change significantly the survival and necrosis rates in PBMC, as revealed by trypan blue exclusion technique (Table II). The supplementary analysis by MTT assay also showed no significant changes with respect to controls in the viability of treated PBMC samples. As for the cell density, determined through quantitative analysis of protein levels, no significant differences were detected between exposed samples and their respective controls (Fig. 7).

### Flow cytometric analysis of the response of PBMC subpopulations to the 50 μA/mm^2^ current

**Figure 8:**
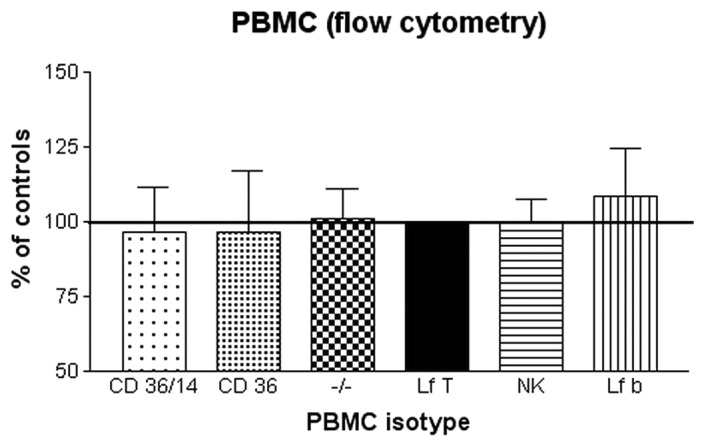
Flow cytometry for PBMC isotypes. Percent of the different isotypes after treatment with 50 μA/mm^2^. CD36^+^/14^+^, CD36^+^ and ^−/−^ (CD36^−^/CD14^−^) are monocyte isotypes; Lf T, T lymphocytes; NK, natural killer cells; Lf B, B lymphocytes. Means ± SD of two experimental replicates. Data are normalized over controls. Differences with respect to controls were not statistically significant.

PBMC populations are composed of cellular subpopulations of different isotypes of monocytes and lymphocytes. In order to study potential treatment-induced alterations in specific cell subpopulations, different cell samples from two healthy donors were analyze by flow cytometry. At the end of the 24 h of intermittent treatment no significant changes were found in the distribution or the relative rate of any of the cell subpopulations (Fig. 8). These results are consistent with those on viability and cell density, and reinforce the indications that PBMC from healthy donors do not respond to subthermal levels of 0.57 MHz electric currents.

## Discussion

Electrothermal CRET therapies induce hyperthermia in deep tissues by transdermal application of electric currents within the 0.45–0.6 MHz frequency range. Pilot trials on patients with glioblastoma multiforme have reported that CRET treatment can reduce peritumoral edema and intratumoral vascularization, and induce extensive necrosis in the tumor core and significant tumor growth reduction, without affecting the surrounding healthy brain tissue (9). More recently, Kato *et al*(22) have shown that CRET treatment administered at thermal doses can significantly potentiate the anticancer effect of 6-O-palmitoyl-ascorbate on human tongue squamous carcinoma cells. Moreover, recent experimental studies by our group have shown that *in vitro* stimulation with subthermal doses of 0.57 MHz, CRET electric currents, induces partial cytostasis in the human hepatocarcinoma cell line HepG2 (16). This cytostasis would be mediated by alterations, also induced by the CRET treatment, in the expression and activation of cell cycle control proteins (cyclins D1, A, B1 and p27), and such alterations lead to a blockage or slowing down of the G_1_ and S phases of the cell cycle of HepG2 (17). Prior, preliminary studies by Hernández-Bule *et al*(15) had reported that the same 0.57 MHz signal administered at non-thermal or subthermal doses, could exert cytotoxic effects in neuroblastoma cells. The results of the present study confirm such cytotoxic response in a fraction of the NB69 population. The effect manifests as significant increases in the rate of necrosis, which at least within the tested density range, are not linearly dependent on the applied current density (Fig. 3A). This reinforces the hypothesis that the cellular response to the electric stimulus is different, and at some extent independent, of the thermal effects attributed to electrothermal therapies.

Multiple cellular pathways leading to cell death have been described, including perturbation of ion homeostasis, activation of proteases and phospholipases, degradation of DNA and generation of reactive oxygen intermediates (23). The molecular basis of the cytotoxic action exerted by CRET currents remain to be elucidated. However, it might be related with electrically-induced alterations in the expression or activation of one or more proteins involved in the cell cycle regulation, as described by Hernández-Bule *et al* and Wang *et al*(17,18).

In addition to the described effects on cell viability, the present results show that intermittent treatment with the 0.57 MHz currents significantly increases DNA levels in NB69, since a significant increase in BrdU incorporation into the DNA was detected at the end of the 24 h of treatment. Additionally, we found a significant increase in the expression of proliferating cell nuclear antigen after 12-h exposure, which appears to last until the end of treatment, even if at 24 h the difference with respect to controls did not reach statistical significance. PCNA is an auxiliary protein of DNA polymerase-δ, which is synthesized during the late G_1_ and S phases of the cell cycle. PCNA expression is correlated with the cell proliferative state and is involved in DNA replication and repair (24–26). In the present study the upregulated expression of PCNA, accompanied with changes in the BrdU staining pattern, indicates that the observed effect in PCNA was proliferative, rather than due to DNA damage. Besides, the amount of energy delivered to the cells by the electric treatment is far too low as to break chemical bonds or cause thermal effects, thus, other alternative mechanisms would be involved in the observed biological responses.

This dual, cytotoxic and cytoproliferative response could be due to alterations in cell cycle progression similar to those reported previously in HepG2 hepatocarcinoma cells exposed to the same electric stimulus (16,17). In fact, the present flow cytometry data reinforce this interpretation. At 6 h of the stimulation onset, modest, non-significant statistical increases were detected in the proportions of NB69 cells in G_0_/G_1_ and, mainly, in S phase. After the first 12 h of intermittent exposure, these increases were followed by a significant decrease, of ∼20% below controls, in the fraction of the cellular population distributed in G_2_ and M phases. At the end of the 24 h of treatment a modest increase in the proportion of cells in S phase was detected, which was similar to that observed at 6 h. Taken together, these results could be indicative of a slowdown or a transient blocking of the S phase in a fraction of the cell population, which would cause cycle elongation and, therefore, the decline in the cell density of the culture, as observed after treatment.

The analysis of the evolution of NB69 cultures showed that the treatment-induced decrease in cell population persisted two days after the end of the electric stimulation (72 h after the exposure onset). Indeed, at that time a 7.59% decrease in the number of living cells and a 17.18% increase in the rate of necrosis, were observed in the exposed samples when compared to controls. Such an effect could result from the combination of a cytotoxic action, exerted on a particularly sensitive cellular subpopulation, plus a transient slowing or blocking of the cell cycle in another subpopulation of the same samples. In fact, it has been reported that the NB69 line integrates two predominant cell subtypes, a subpopulation with 45 chromosomes and another with 48 chromosomes, which show a marked tendency to undergo cytogenetic changes (27). These subpopulations could respond differently to the electric stimulus. In this context it must be underlined that at the end of the 24 h of intermittent stimulation with current densities j <400 μA/mm^2^, the cell death rates were significantly increased with respect to controls, whereas the number of living cells did not change significantly (Fig. 3B). This could be attributable to the existence of a third cell subtype, recently described by Acosta *et al*(28). This subpopulation could either be insensitive to the electric stimulation or respond to it by slightly increasing its proliferation, which would partly compensate the decline in the viability rate due to the treatment-induced necrosis in the vulnerable subpopulation. However, since in general the samples treated with j ≤400 μA/mm^2^ tend to show a decrease in the total number of viable cells (Fig. 3B), it is conceivable that in our cultures the relative size of the third subpopulation would have little relevance.

Within the context of the existing evidence on the cellular response to subthermal, 0.57 MHz electric currents (16,17), the analysis of the present data indicates that at least a fraction or subpopulation of the NB69 cell line, would be more responsive to the electric stimulus than the HepG2 cells. Such differential sensitivity could be related to the proliferation rate, which is higher in NB69 (doubling time = 12 h in our experimental conditions) than in HepG2 (doubling time = 42 h). This hypothesis would be supported by the lack of responsiveness of PBMC cultures, which are virtually quiescent unless activated by exogenous mitogenic agents. Indeed, our data show that the same electric stimulus that elicits significant changes in the cell cycle, cell proliferation and the viability of two human tumor lines, does not affect any of the studied processes in PBMC. Collectively, these results are consistent with those reported by other authors showing that dividing cells are particularly, if not exclusively, sensitive to the effects of electric stimuli of frequencies ranging from 100 KHz to 1 MHz (4,19).

In conclusion, our results show that human neuroblastoma cells NB69 are sensitive to the *in vitro* exposure to short pulses of 0.57 MHz subthermal currents applied over a period of 24 h. This response is similar in nature to that reported in the line HepG2 when exposed to the same electric stimulus. This reinforces the evidence that at least certain types of tumor cells are sensitive to 0.57 MHz currents, and that the cellular response involves mechanisms other than those triggered by conventional thermal stimuli. In connection with electro-thermal CRET treatments applied to cancer patients, it has been proposed that the partial cytotoxic response observed *in vitro* in NB69, or the cytostatic effect reported in HepG2, could act in synergy with the potential oncostatic action exerted by the hyperthermia induced in the tumor tissue by Joule's effect (15). The fact that PBMC from healthy donors have been revealed irresponsive to the electric stimulus, strengthens the hypothesis that non-proliferating cells, or cells with low proliferation rates, do not respond to the electric treatment. This could be envisioned as an endorsement to the current medical evidence on safety of therapies based on the application of the tested stimulus. Thus, the available experimental evidence on human cellular models is supportive of the claim that CRET could be effective as a potential adjuvant in the treatment of some cancer related processes. However, a deeper understanding of the cellular and molecular mechanisms of response to electrical stimuli in the intermediate frequency and radiofrequency ranges, is crucial to the potential applications of electro-thermal therapies like CRET in oncology.

## Figures and Tables

**Table I:** Treatment-induced changes in the cell cycle of NB69.

Time (h)	No. of experimental replicates	% G_0_/G_1_ phase	% S phase	% G_2_/M phase
6	2	108.9±4.4	115.2±15.9	111.9±27.3
12	2	103.7±5.5	101.0±14.4	82.1±2.3^a^
24	3	97.6±9.8	111.1±17.0	107.1±24.5

Time course of the cell distribution in different cell cycle phases during (6 and 12 h) and end (24 h) of treatment of NB69 cells with 0.57 MHz currents. Flow cytometry with propidium iodide. Values are percentages of the respective controls and represent means ± SEM of two or three experimental replicates as shown in Fig. 6. Student's t-test;

a 0.01< p<0.05.

**Table II:** PBMC viability before and after treatment.

Time after seeding (h)	Electrodes present	Current density (μA/mm^2^)	Viable cells per dish (n)	Necrotic cells per dish (n)
0	No	0	11.0x10^6^±0.0	1.0x10^3^ ±0.0
1	No	0	10.9x10^6^±0.0	30.0x10^3^±3.0x10^3^
24	Yes	0	7.2x10^6^±0.4x10^6^	254.2x10^3^±70.2x10^3^
24	Yes	50	7.9x10^6^±0.6x10^6^	302.1x10^3^±17.8x10^3^

Viability of PBMC as a function of the incubation time and of the electric treatment. Trypan blue dye exclusion method. Electrode pairs were inserted into all Petri dishes at the end of the first hour of incubation post-seeding, after the count of living and necrotic cells. Samples treated with 50 μA/mm^2^ were intermittently exposed until the end of the 24 h post-seeding. Means ± SEM of ten experimental replicates. Differences with respect to controls were not statistically significant.
